# Advances in mass cytometry and its applicability to digital pathology in clinical-translational cancer research

**DOI:** 10.1515/almed-2021-0075

**Published:** 2021-11-24

**Authors:** Karina Cereceda, Roddy Jorquera, Franz Villarroel-Espíndola

**Affiliations:** Laboratorio de Medicina Traslacional, Instituto Oncológico Fundación Arturo López Pérez, Santiago, Chile

**Keywords:** imaging mass cytometry (IMC), multiplexed ion beam imaging (MIBI), oncology

## Abstract

The development and subsequent adaptation of mass cytometry for the histological analysis of tissue sections has allowed the simultaneous spatial characterization of multiple components. This is useful to find the correlation between the genotypic and phenotypic profile of tumor cells and their environment in clinical-translational studies. In this revision, we provide an overview of the most relevant hallmarks in the development, implementation and application of multiplexed imaging in the study of cancer and other conditions. A special focus is placed on studies based on imaging mass cytometry (IMC) and multiplexed ion beam imaging (MIBI). The purpose of this review is to help our readers become familiar with the verification techniques employed on this tool and outline the multiple applications reported in the literature. This review will also provide guidance on the use of IMC or MIBI in any field of biomedical research.

## Introduction

Considerable efforts have been made to try to integrate the different sources of information about tumor biology, such as morphology and genetic features [1], [2], [3], [4]; however, technology and classical tools have failed to combine the multiple biomarkers that could be potentially useful for cancer staging or patient-treatment matching, or identifying the correlation between treatment and the expected clinical outcome.

Tumor microenvironment is defined as the extracellular environment and cellular components of the affected organ, surrounding tumor cells. This environment consists of neoplastic and non-neoplastic cells such as fibroblasts, lymphocytes, macrophages and other immune cells, as well as vascular components, and the extracellular matrix itself, including its fluids and dissolved molecules such as secreted cytokines, chemokines, metabolites, and extracellular vesicles [5], [6], [7]. There is cumulative evidence demonstrating that non-neoplastic cells residing in tumor-adjacent tissues may play an active role in the neoplastic process and even facilitate the evasion of tumor cells from the immune system [7]. In this scenario, tumor microenvironment may promote malignant transformation and progression, metastasis and even resistance to conventional and emerging therapies [6, 8].

Over the years, health problems in developed countries have changed as a result of the aging of the population. Thus, chronic multimorbidity, as well as cancer, has become increasingly frequent.

Evidence-based research and technological advances have pushed modern health centers to progressively adopt a holistic, patient-centered care approach [9]. Such advances have also facilitated patient stratification based on enhanced diagnostic tools and on the use of instruments predicting therapy success, especially in cancer patients.

According to the American Society of Clinical Oncology (ASCO), “panomics” is defined as the integration of clinical data with data obtained from analytical or “omic” platforms [10]. This integration strategy has been identified as one of the driving factors that will model the approach to cancer by 2030 [11].

In the field of oncology, the integration of “omics” [12, 13], data from electronic clinical records [14] and, more recently, “radiomics”, digital pathology, bioinformatics, and artificial intelligence [15], among other technological advances, make it possible to stratify patient populations into subpopulations, based on their risk to develop a given condition or respond to a particular therapy.

## Multiple component imaging and mass spectrometry

Recent advances in multiplexed single-cell spatial analysis of tissue specimens have uncovered the complex biology of cancer at an unprecedented resolution [16]. Cutting-edge technology makes it possible to identify the type and status of cells in a tumor and accurately determine their spatial location within the tumor. The combination of these techniques with computational biology will help us better understand tumor progress and heterogeneity in its microenvironment and build models that capture the complexity of *pan-omics* data in different patients. Ultimately, they will allow us to fine-tune diagnostics and therapeutics [11, 17, 18].

The heterogeneous response of cancer patients to immunotherapy highlights the need to better understand tumor microenvironment [19] and cell interactions within a tumor. This need will expectantly drive the development of new technologies and methods that provide *omics* data and multiplexed images of tissue specimens [20], ultimately leading to the identification of potential therapeutic targets [17, 21, 22].

The intensive use of immunohistochemistry (IHC) and imaging techniques in the clinic demonstrates the relevance that spatial information has for digital pathology and clinical-translational research.

In clinical practice, the standard protocol for cancer diagnosis includes the immunohistochemical analysis of a stained paraffin-embedded tissue specimen on a microscope slide [23, 24].

Limitations of conventional IHC include high inter-observer variability [25] and the fact that tissue sections can only be stained with two or three markers at a time. This way, to study various biomarkers, it is necessary to analyze multiple tissue sections through serial histological sectioning, which is challenging when only a small sample of tissue is available [24]. This approach limits the possibility of obtaining a general picture of a complex immunological microenvironment. For example, IHC is not useful to characterize subsets of CD8-positive T-cells with different functionalities infiltrating the tumor nest (apoptotic, proliferative, cytotoxic, memory T-cells, to name a few). Also, IHC is not effective in identifying, simultaneously, lymphocyte populations such as CD8, CD4, and CD20 in a region of interest [24, 26]. In addition, in a variety of tumors, patient-treatment matching is performed on the basis of the expression of specific molecules such as PD-L1 on tumor cell surface [27, 28]. The presence of lymphocytic inflammatory infiltrate and PD-1 overexpression on the surface of CD8-positive T-cells has been largely proven to predict response to anticancer immunotherapies inhibiting PD-L1/PD-1 binding [22]. These markers may have predictive value either alone or in combination. Some researchers support their combined use, as they have been proven to have prognostic value in several types of cancer [20, 22].

A variety of IHC techniques have been developed to stain and visualize a larger number of molecules in a single tissue section. These techniques, commonly known as multiplexed IHC, combined with the use of fluorescent-labeled secondary antibodies, have made an undeniable contribution to scientific knowledge. IHC, however, has significant limitations, such as spectral interference when more than one fluorescent label is used, cross-reactivity between antibodies, photo-bleaching, fading of the fluorescent dye, and autofluorescence of the tissue [29].

Several cutting-edge methods have the ability to detect multiple components, albeit with lower sensitivity, resolution, or accessibility [30]. Table 1 summarizes the different multiplexed imaging technologies and procedures applied to formalin-fixed tissue sections.

**Table 1:** Characteristics, advantages, and disadvantages of platforms based on multiplexed detection technology (>10 targets).

Method/technology	Authorship	Type of tissue	Analyte	Limit of detection	Multiplexing	Resolution	Advantages	Disadvantages	Description
Multiplexed fluorescence microscopy (MxIF)	Not private [31]	FFPE tissue [31]	Proteins and RNA [35]	Fluorescence [35]	61 proteins [31, 35]	Subcellular, ∼1 μm [35]	Generation of multiplexed images [35]	Re-alignment of images acquired in each image acquisition cycle [35]	Method of multiplexed fluorescence microscopy (MxIF) for the quantitative, single-cell and subcellular characterization of multiple analytes in FFPE tissue. The chemical inactivation of fluorescent dyes after each image acquisition cycle in iterative staining and imaging cycles [31].
Single-cell gene expression (10X Chromium)	10× Genomics	FFPE or frozen tissue [19]	RNA [19]	DNA barcoding and NGS [19]	Thousands of reads	Cellular [19]	Whole transcriptome [19]	No bidimensional resolution [19], no multiplexed imaging	Method combining the analysis of single-cell gene expression with high-resolution detection of hundreds of cellular surface proteins for multi-omic cytometry (product sheet).
Spatial gene expression (10× Visium)	10× Genomics	FFPE or frozen tissue [19]	RNA [19]	DNA barcoding and NGS [19]	Thousands of reads	Cellular, 55–100 μm [19]	Whole transcriptoma [19] generation of multiplexed images	Different types of cells can be detected in a bar-coded region [19] Low resolution	Visium technology combines gene expression analysis by staining and image analysis by immunofluorescence staining to obtain a multi-omic characterization within a given positional context (product sheet).
InsituPlex	Ultivue	FFPE or frozen tissue [19]	Proteins and RNA [20]	DNA barcoding and fluorescence [19]	16 proteins [20]	Sub-cellular [19]	Generation of multiplexed images [19]	Non-compatible with automated slide scanners [19]	InsituPlex technology relies on the amplification and bar-coding of DNA conjugated to primary antibodies to provide bidimensional multiplexed images in whole slides [19].
Co-detection by indexation (CODEX)	Akoya	FFPE or frozen tissue [19]	Proteins [19]	DNA barcoding and fluorescence [19]	50 proteins [16, 32]	Subcellular, ∼260 nm [20]	Generation of multiplexed images [19]	Can be extremely time-consuming [19]	Antibodies conjugated to single oligonucleotide sequences that are detected cyclically by the sequential primer elongation with fluorescently-labelled nucleotides [16].
Digital spatial profiling (DSP)	NanoString	FFPE or frozen tissue [19]	Proteins and RNA [19, 35]	Bar-coded DNA and ultraviolet light [20, 35]	40 proteins or more than 90 RNAs [16, 35]	Cellular, ∼10 μm [19, 35]	Whole transcriptoma and high level of automatization	No generation of multiplexed images [19]. It is time-consuming and has a low resolution [16, 28]	RNA labelling with antibodies or RNA probes with photoresponsive DNA oligonucleotides that are released and quantified after a specific tissue region is exposed to ultraviolet light [10, 16].
Multiplexed ion beam imaging (MIBI)	IonPath	FFPE tissue [37]	Proteins and RNA [35]	Lanthanide metals and mass spectrometry [35]	36 proteins [35, 47]	Subcellular, ∼200 nm [35]	High-resolution multiplexed imaging [16]	Expensive and time-consuming [16]	Method combining antibodies conjugated to lanthanide isotopes and detection by a mass spectrometer fitted with a supply of oxygen ions [16, 47].
Imaging Mass cytometry (IMC)	Fluidigm	FFPE or frozen tissue [19]	Proteins and RNA [35]	Lanthanide metals and mass spectrometry [35]	32 proteins [35]	Subcellular [19], ∼1 μm [35]	Multiplexed imaging [35]	Expensive and time-consuming [16, 41]	Technology based on the ablation of a tissue section with a pulsed laser. Antibodies conjugated to lanthanide isotopes detected by mass spectrometry [16, 36].
Immunostaining with signal amplification by exchange reaction (Immuno-SABER)	Not private [30]	FFPE or frozen tissue. Cellular preparations [30]	Proteins [30]	DNA-barcoded antibodies and fluorescence [30]	10 proteins [30]	Subcellular, ∼160 nm combining expansion microscopy [30]	Compatible with several specimens and platforms. High-resolution multiplexed imaging [30]	Fluorescence signal dilution when combined with expansion microscopy [30]	Multiple DNA-barcoded primary antibodies are hybridized to single-stranded DNA concameters generated via primer exchange reactions [16].
Slide-seq	Not private [34]	FFPE or frozen tissue [34]	RNA [34]	Bar-coded DNA and NGS [34]	Thousands of transcripts [34]	Cellular, ∼10 μm [34]	Whole transcriptome associated with coordinates on tissue [16]	Low resolution	RNA is transferred from tissue sections onto a surface covered in DNA-barcoded beads with known positions, allowing the spatial locations of the RNA to be inferred by sequencing.

FFPE, formalin-fixed paraffin-embedded; IMC, imaging mass cytometry.

Some of these novel methods are based on cyclic immunofluorescence [31], the use of oligonucleotides as bar codes [19, 30, 32], [33], [34], [35], mass spectrometry combined with classic histological techniques and targeted mass spectrometry based on labeled antibodies [36, 37]. These instruments provide a higher phenotypic specificity, as compared to classic IHC. In addition, they allow optimizing specimen use by detecting multiple markers at a time in a single sample section. These instruments also increase the sensitivity of methods through the combined use of several markers for a specific type of cell.

**Figure 1:**
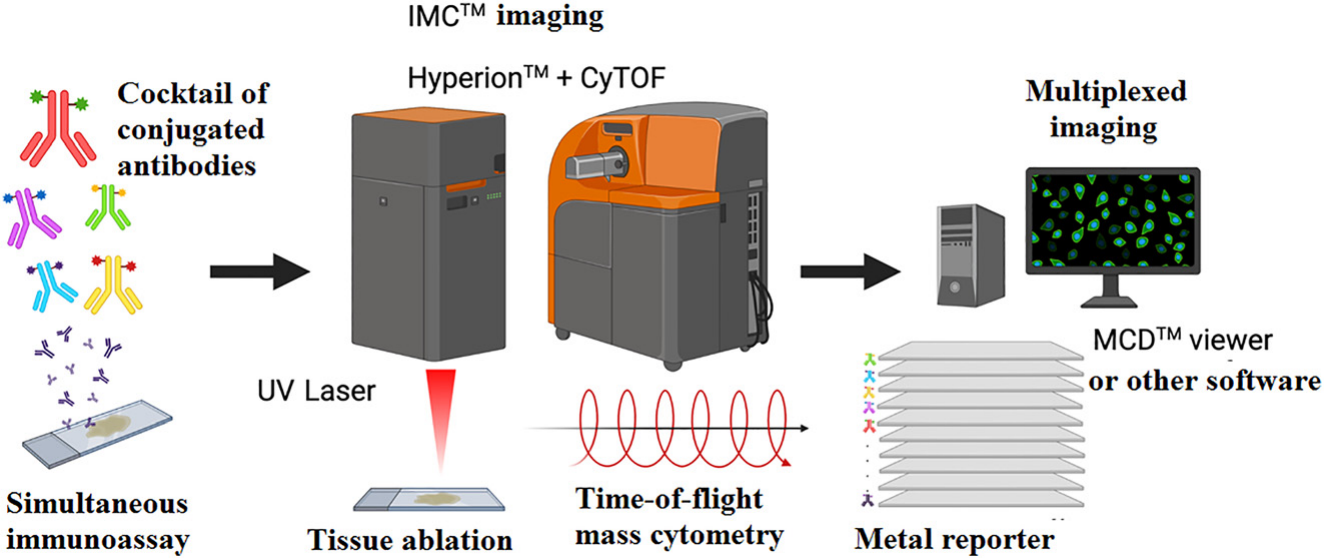
Workflow of imaging mass cytometry (IMC™). Histological tissue sections or immobilized cells are simultaneously immunodetected with multiple antibodies conjugated to different isotopes (reporters). After tissue ablation by pulsed laser on Hyperion™, the vaporized sample is driven to the mass detector of the mass cytometer (CyTOF^®^) through a plasma flow of inert gas. A set of coordinates allows reconstructing the tissue and the association with the abundance of each reporter. Different tools enable signal (image) analysis and overlapping. Figure designed by https://app.biorender.com/.

Imaging mass cytometry (IMC) yields images through time-of-flight analysis of rare metal isotopes conjugated to a specific antibody. This way, drawing on the principle described for mass cytometry, each antibody acts both as detector and reporter [38] (Figure 1).

Multiple targets can be detected through the presence of stable isotopes from the lanthanide series, which are chelated using a synthetic polymer containing functional groups 1,4,7,10-tetraazacyclododecane-1,4,7,10-tetraacetic (DOTA) or diethylene triamino pentaacetic (DTPA), which are conjugated to the heavy chain of immunoglobulin G (IgG) through activated sulfhydryl groups. Near 40 isotopes are widely employed, which are resolved with a precision of a unit of atomic mass in more than 95% of cases, and for which there is an optimized conjugation protocol [39].

From the point of view of imaging, IMC technology permits the ablation of tissue or immobilized cells on a conventional silicate glass using a pulsed laser operating at 213 nm and focused to a 1 µm diameter spot size. The sample is vaporized on each laser shot and carried into the mass cytometer by the stream of plasma of an inert gas. Apart from the cloud of atoms, each laser shot generates a set of coordinates that allows us to reconstruct the tissue and associate it with the abundance of reporters in each event [38].

MIBI enables the characterization of histological sections based on metal-labeled antibodies [37]. It is very similar to IMC but ablation is performed under different conditions. Although MIBI uses a beam of oxygen ions in a vacuum chamber, IMC employs a laser in a chamber at atmospheric pressure. In general, the two technologies seem to be comparable in terms of sensitivity, resolution, and complexity of results [40]. In some cases, when combined with other technologies such as transcriptomic analysis at single-cell resolution, these methods might provide valuable information about the biological complexity of tumor microenvironment.

A review is provided below of the main hallmarks in the development of multiplexed imaging both, by IMC and MIBI, and its application to clinical-translational research.

## Timeline of the analysis of multiplexed images

The information provided by imaging mass spectrometry has made it possible to develop therapeutic and diagnostic models that could help classify patients based on tumor cell phenotype and genotype or on the architectonic features of neighboring structures. Different scientific, academic and clinical groups are currently using these tools to perform tissue segmentation and phenotypic profiling at diagnosis, start of therapy and throughout the treatment (Table 2).

**Table 2:** Summary of studies on multiplexed imaging based on the use of metal reporters and mass cytometry.

Type of study	First author	Disease/tissue of interest	Number of markers	Target
Technique optimization	Angelo, Michael [37]	Breast cancer	10	Validation of MIBI for FFPE specimens with clinical application.
	Gerdtsson, Erik [42]; Bath, Izhar [61]; Giesen, Charlotte [36]	Different types of tumors	18–32	Validation of IMC for differentiating specific cell types.
	Martinez-Morilla, Sandra [57]	Melanoma	26	Validation of “AQUA” for IMC analysis. Identification of 10 candidate biomarkers responsive to immunotherapy- confirmation of B2M as a survival-associated target.
	Schulz, Daniel [44]	Breast cancer	3 ARNm, 16 proteins	Validation of simultaneous detection of nucleotides and proteins by IMC in single-cell assays. Determination of a high correlation between ARNm and protein levels for HER2, but not for CK19. CXCL10 expresses in stromal cell clusters and is correlated with the presence of T-cells.
	Guo, Nannan [62]	Fetal and adult intestinal tissue	34	Development and validation of a panel of 34 antibodies for the analysis of IMC of frozen specimens.
Characterization of tumor microenvironment	Carvajal-Hausdorf, Daniel [52]; Rost, Sandra [63]	Breast cancer	3 to 18	Characterization of HER-2 levels and functions in breast cancer.
	Keren, Leeat [47]; Ptacek, Jason [60]	Different types of tumors	15 to 36	Expression and location of different immune regulatory proteins.
	Ijsselsteijn, Marieke E [46]	Colorectal cancer	40	Description of a panel of antibodies for monitoring tumor immune microenvironment by IMC.
	Li, ran [64]	Squamous cell carcinoma (lung)	21	CD45RO+CD8+ T-cell infiltration; Description of a new population of CD3-CD4+ T cells in tumor immune microenvironment (TIME).
Digital pathology miscellaneous	Singh, Nikhil [49]	Kidney	23	Development of an atlas of kidney tissue markers. Identification of potential new types of cells that enable differentiation between normal and abnormal tissue.
	Theil, Diethilde [48]	Lymphoid nodules in Cynomolgus monkeys	11	Characterization of lymphocyte subpopulations. Treatment with ofatumumab generates a rare population of T-cells that are CD3+, CD8+, CD20+, located in peripheral B-cell follicles.
	Wang, Chong [65]	Lung, small-bowel, spleen, liver, and kidney of patients who died from COVID-19	23	Infiltration of CD11b+ macrophages and CD11c+ dendritic cells in lungs and small bowel: High IL-10 levels in lungs and small bowel; TNF-α overproduction in the lungs, small bowel, kidneys and spleen.

B2M, beta2-microglobulin; CK19, cytokeratin 19; FFPE, formalin-fixed paraffin-embedded.

## Mass cytometry in histology

In 2014, Giesen et al. reported the usefulness of cytometry by time-of-flight (CyTOF) for the analysis of suspended tissue specimens to acquire multi-parametric images using CyTOF technology. This study was performed in malignant breast lesions and their respective non-tumor controls, where each antibody was covalently bounded to the detection metal isotope to analyze 32 proteins separately at subcellular resolution. Building a high-dimensional image involves overlapping the flight times of each reporter with the coordinates of the ablation laser. This way, a multi-dimensional and multi-parametric composition is obtained with the spatial resolution required to perform segmentation and phenotyping studies [41]. The authors previously assessed the concordance between staining patterns and signal intensities for each antibody by classic immunodetection, and defined variations between metal-conjugated antibodies and unconjugated antibodies.

Signal intensity variability ranged between 2–27%, but no variability was observed in staining patterns. This difference was attributed to intrinsic variation among staining agents in serial non-identical tissue sections [36]. Computational analysis and bioinformatics demonstrated that imaging mass cytometry (IMC) was highly sensitive, reproducible, and comparable to the images obtained by conventional IHC, immunocytochemistry, and immunofluorescence and, in addition, make it possible to distinguish the structure and features at the level of single-cells and from whole-tissue sections.

The use of IMC for the analysis of individual cells’ spread after the publication of the study by Gerdtsson et al. [42], who integrated high-definition single-cell analysis (HD-SCA) [43] and IMC. This technique enabled the morphological analysis and phenotyping of single rare cells, circulating tumor cells, disseminated tumor cells, and minimal residual disease. Based on human cancer-derived cell lines (LNCap in prostate and MDA-MB-231 in breast cancer), the authors established the conditions of sensitivity, specificity, and linearity for the detection of these cellular fractions within a human whole-blood matrix. The tumor cell is identified in the leukocyte smear by fluorescent staining with cytokeratin and CD45; then, labeling with metal reporters is performed using the battery of selected antibodies. Based on the spatial coordinates previously obtained by laser ablation, the authors acquired 400 × 400 um images of the region of interest with a resolution of 1 um^2^ using a laser pulse of 200 Hz. With a relatively small antibody panel, the quality of the image obtained by IMC allowed the authors to individualize neoplastic cells at a relative abundance of one per 10,000 nucleated blood cells. Notably, owing to the signal-to-noise ratio and limit of detection established by the authors, historical samples could be re-analyzed and compared with previously reported findings, with a high level of concordance [42].

Due to their versatility, IMC reporters are used for the detection of oligonucleotides and nucleic acids. In the past, nuclear components could be evaluated using DNA intercalating agents or by the detection of some histone. However, Schulz et al. [44] suggest that messenger RNA (mRNAs) and protein levels can be determined in histological samples at the same time. The main contribution of these authors was that they modified the RNAscope^®^ protocol [45] for *in-situ* hybridization and replaced the detection probe with an oligonucleotide conjugated to a metal reporter for IMC staining. Once hybridization is completed, the tissue or specimen was ready for later processing, including immunodetection for IMC.

To validate this novel application, the authors assessed concordance between fluorescence *in-situ* hybridization (FISH) and the signal from the metal reporter conjugated to the probe, considering the detection of mRNA of constitutive genes (POLR2A, PPIB, and UBC) in paraffin-embedded HeLa cells. The correlation between detection methods ranged from 0.89 to 0.8 [44].

In very general terms, the ability of this new method to detect nucleic acids and proteins simultaneously was verified in 70 patients with breast cancer considering 16 target proteins and three target RNAs. To such purpose, the authors assessed the correlation between the abundance of proteins and mRNAs for human epidermal growth factor receptor 2 (HER2) and cytokeratin 19 (CK19). However, results were not conclusive, and it could be attributed to some regulatory mechanism other than those addressed in the experiment. In any case, this technique allows the detection of mRNA and proteins at a time, providing a detailed cellular, phenotypic and functional characterization of single-cells in FFPE by IMC.

Finally, from a methodological point of view, several authors have generously shared their experiences and published their results. A recent paper summarizes antibody staining and handling conditions for a panel of 40 biomarkers, with special relevance given to antigen recovery conditions. The authors compared the pH of the recovery buffer at standard temperature and pressures, and found that for their selected antibodies, a low pH (10 mM Citrate pH = 6) would have a favorable effect, as compared to a high pH (10 mM Tris/1 mM EDTA pH = 9), based on individual IHC results and detection by DAB staining. After having evaluated 65 antibodies, Ijsselsteijn et al. [46] selected 40 monoclonal antibodies and reported their conjugated versions, incubation conditions and working dilutions. Unfortunately, the authors did not report the concentrations of each stock, which prevent homologation. However, this explanatory and detailed paper is very useful when it comes to design an IHC protocol for IMC.

## MIBI, a new kid on the block

Among IMC tools, the one designed by Angelo et al. [37] and later improved with the integration of time-of-flight analysis [47] has been proven to be highly reproducible and reliable for the analysis of fresh and paraffin-embedded tissue specimens. The first experiences with multiplexed ion beam imaging (MIBI) were based on the use of peripheral blood mononuclear cells (PBMC), which were labeled in suspension using classic surface markers such as CD3, CD4, and CD8 for T-lymphocytes, CD14 mainly for monocytes, CD19 for B-lymphocytes, and generic markers for immune cells CD45 and HLA-DR. Two identical fractions were generated, a cellular fraction was embedded in silicone for MIBI, whereas the other was processed for mass cytometry (CyTOF). The analysis showed a high correlation between the two tools in signal intensity and absolute count of the elements detected for each category, with a dynamic range of 10,000 counts for MIBI and a variance <1% between mass cytometry and imaging analysis. In addition, based on the staining strategy employed, MIBI may allow for the analysis of isolated cells and/or cells grown in suspension without needing a smear or imprint; this technique offers the possibility of centrifuging and embedding cells in silicone or other compatible substrate. In this context, FFPE tissues have also been analyzed by MIBI in accordance with the following method: 5 um-thick samples were directly labeled with initial combinations of 10 antibodies [37], then the panel was progressively increased up to 40 [47].

It is worth mentioning that the results of the histological analyses performed on MIBI images were consistent with those of classic IHC tools. Angelo [37] demonstrated that the specificity of the primary antibody is maintained after it is modified chemically, with a signal intensity and noise comparable to those of chromogenic staining. In addition, automated quantitative image analysis based on bioinformatics was proven to be compatible, with a high concordance with the validated diagnostic tools currently available (for example QIA, quantitative image analysis FDA-approved), with an H score of 1.06.

In accordance with different authors, MIBI has multiple advantages over conventional IHC. (i) Higher sensitivity and analytical dynamic range, increasing 100 and 1,000 times signal-to-noise ratio, as compared to fluorescence and chromogenic staining, respectively; (ii) higher specificity, albeit it depends on the primary antibody employed. The high analytical resolution of mass analysis (mole Dalton’s fraction) prevents spectral overlapping between adjacent reporters and the multiplicity of detectable targets; (iii) an optimized use of scarce biological material. Reporters are highly stable in time, and samples can be repeatedly scanned at different resolutions (from 260 nm to 1 um).

More recently, MIBI demonstrated to be useful in the study of triple-negative breast cancer with a large panel of 36 target proteins. This technique has revealed that some of the phenotypic characteristics of the tumor are related to the architecture of the tissue and its neighboring environment [47]. Some authors describe a heterogeneous distribution of positive PD-L1 tumor and non-tumor cells within and between subjects. Researchers have also observed a high abundance of positive HLA-DR cells in tumor margins and stromal area, which could be associated with higher survival rates [47].

## Innovative applications

### Non-human models

IMC has shown an unexpected versatility, having been used not only in human but also in animal tissue. Considering all guidelines for the validation of antibodies and the conditions for immunodetection described above, Theil et al. [48] established a panel of nine antibodies to characterize lymphocyte subgroups in blood and lymphoid tissue in Cynomolgus monkeys, who received Ofatumumab at doses equivalent to those employed in humans [48]. The IMC technique has shown high plasticity and is gaining ground in studies with a higher impact on human health. Ofatumumab is the first anti-CD20 monoclonal antibody to be tested in a phase III trial for the treatment of multiple sclerosis.

### Physiopathology of kidney disease

Efforts to reproduce the complexity of healthy and unhealthy human tissue have intensified significantly in the recent years. Singh et al. [49] used a set of 23 antibodies to create a 2-dimensional atlas of the human kidney at a very high resolution level, exploring the tissue from the cortex through the renal medulla. The high sensitivity, specificity, and multiplicity of signals enabled the authors to characterize transitional tubular regions, which had never been described in such detail before [49]. Additionally, the authors analyzed pathological tissue and paid special attention to populations of infiltrating immune cells in a setting of kidney transplantation and nephritis. The validation of antibodies and protocols was shared on (Re)Building a Kidney (https://www.rebuildingakidney.org/) [50].

### HER2 and trastuzumab

From the point of view of cancer research, IMC and other technologies have made a valuable contribution to knowledge, especially regarding the development of biomarkers. At present, trastuzumab is a therapeutic antibody widely used in breast cancer in which binding site is located at the extracellular domain (ECD) of HER2 [51]. One of the advantages of IMC is that it allows the evaluation of a single marker from two perspectives. Carvajal-Hausdorf et al. [52] included two anti-HER2 antibodies in the same panel in which specificities were directed towards an epitope on the ECD and an epitope on the intracellular domain (ICD). After a retrospective analysis of breast cancer biopsies, the authors found that the specimens of the patients who experienced recurrence after adjuvant trastuzumab were rarely HER2 ECD-positive. Clinically, an elevated ECD/ICD ratio would be associated with a recurrence-free survival >5 years and with a significant abundance of positive CD8 cytotoxic lymphocytes in neighboring tissues. Although only 16 antibodies were tested in that study, the orthogonal validation performed by Carvajal-Hausdorf et al. added to previous evidence on the role of HER2 domains reported by the same group, suggesting that simultaneous classic immunodetection of one or two markers is useful after the variables with a lower significance have been excluded.

### Strategies of analysis

With regard to image analysis strategies, most authors used Fluidigm MCD™ viewer [53]. This application identifies the levels detected by each metal reporter and provides its geographic location in the tissue. However, the most relevant primary and secondary analyses were performed using the tools described by Dr. Bernd Bodenmiller, from the University of Zurich in Sweden [54], [55], [56]. These are open access applications that allow for signal quantification and segmentation into tissue areas, identification of attributes, and phenotyping, for an appropriate multi-parametric analysis.

Recently, the group led by David Rimm at Yale University, USA, reported the use of AQUA™ (Navigate BioPharma Inc) for the primary analysis of IMC images [57]. This group is highly experienced in the use of this software for quantitative fluorescence analysis, where tumor regions are identified using a mask associated to a pixel [58, 59]. Martínez-Morilla et al. analyzed malign melanoma lesions using AQUA™, which takes pixels from the reactive areas for the DNA intercalator (Ir191/193) and for HMB45+S100 to generate masks for tissue and tumor, respectively. Likewise, the authors performed digital profiling of tissue and other cells, based on the density of pixels for each reporter. Initially, the authors included 26 antibodies; however, this simplified method of analysis enabled the authors to identify 12 markers significantly associated with progression-free survival and only seven antibodies were related to overall survival. The role of beta-2-microglobulin as a biomarker of survival in patients with metastatic melanoma and immunotherapy has been validated by mRNA analysis and simple fluorescence detection; therefore, this protein emerges as a promising biomarker [57].

At present, other private actors have contributed to the development of user-friendly tools such as those developed by Indica labs (HALO^®^) and Visiopharm^®^.

### Validation of multiple components

Other researchers recently assessed the capacity of MIBI to analyze multiple types of solid tumors in different organs and tissues. The study considered 15 types of tumors, including adenocarcinoma, squamous carcinoma and hematologic neoplasms [60]. As their predecessors, the authors evaluated the specificity and sensitivity of their panel of antibodies by IHC, considering endogenous positivity and negativity controls within the same section of tissue. A panel of 15 antibodies was used for immunodetection in 1 mm tissue sections. Images were acquired in optic fields of 0.25 mm^2^ (0.5 µm per pixel). A contribution of these authors is that they applied a simple leave-one-out (LOO) statistical method to verify the signals obtained and avoid contamination between neighboring reporters. Staining was successively repeated after a specific antibody was left out from the panel. The results obtained with the entire panel (15 antibodies) were compared with those after an antibody was left out (14 antibodies). After comparison of eight panels, linear regression analysis was performed, with a correlation R2 = 0.99–1.00, which indicates no interference across signal sources. Based on this premise, the authors carried out tissue segmentation and cellular phenotyping in each of the tumors selected. The results confirmed the high heterogeneity of the components of immune infiltrate, both in terms of abundance and distribution not only across patients with different types of tumors but also with the same type of lesion [60].

## Conclusions

To decide which technique is the most appropriate in each case, specialists must be aware of the advantages and disadvantages that IMC (including MIBI) has over classic IHC considering their shared features and the fundamentals of multiplexed imaging detection (Table 3).

**Table 3:** Advantages and disadvantages of tools for analyzing biomarkers on formalin-fixed paraffin-embedded tissue samples.

Tool	Use of tissue		Potential targets		Reporters	
	Advantage	Disadvantage	Advantage	Disadvantage	Advantage	Disadvantage
Classic immunohistochemistry	Preserves the slide for multiple reads	Multiple slides for complex studies	From 1 to 2 targets (plus nuclear contrast). The marker can be analyzed by multiple observers.	Restricted number of combinations due to the method of detection and location of the marker.	Mostly, insoluble chromogenic deposits stable in time.	Limited number of options (enzymes, conjugates, and chromogenic substrates)
Multiplexed fluorometric immunoassay	Requires a lower number of slides in complex studies	Slides can be preserved for further reads	Up to 9 targets depending on the platform. Allows multiple signals from similar locations.	Requires thorough validation, expensive validation.	Multiple combinations based on the supplier. Allows the use of signal amplifiers.	Risk of photo-bleaching and spectral overlapping of close fluorescent signals.
Multiplexed immunohistochemistry	Requires a lower number of slides in complex studies	Tissue destruction. A second read is rarely possible	Up to 60 targets based on the platform. Allows multiple signals from similar locations.	Requires thorough, expensive validation.	Multiple combinations based on the supplier. Possibilities of reporters overlapping is limited.	Commercially available or prepared at the lab. Expensive reagent. Limited use of signal amplifiers.

Nevertheless, both IMC and MIBI allow for the acquisition of complex images with spatial resolution, and the quantification of multiple components at the same time. It is worth noting that the two approaches are similar to classic histological techniques in terms of specificity and sensitivity. From the point of view of quantification, precision and reproducibility, IMC and MIBI have shown to be comparable and robust in different matrices and targets, regardless of the complexity or simplicity of the antibody panel.

## Perspectives

It is necessary to determine the abundance and spatial distribution of biomarkers to better understand the physiological processes that occur in normal and tumor tissue. In complex diseases such as cancer, being aware of the correlation between the genotype and phenotype of the different cell components and neighboring structures is crucial to make therapeutic decisions, measure clinical benefit, and reduce therapeutic failures. The acquisition of multiplexed images by IMC, MIBI, or other tools has been extensively validated and verified by different researchers, although a technique of reference is necessary to anticipate deviations. This review is intended to provide an overview of the technologies currently available for research, based on a thorough selection of the literature to reduce the learning curve and facilitate the achievement of results.
